# Atractylodin Ameliorates Colitis via PPARα Agonism

**DOI:** 10.3390/ijms24010802

**Published:** 2023-01-02

**Authors:** Gwangbeom Heo, Yuju Kim, Eun-La Kim, Soyeong Park, Sang Hoon Rhee, Jee H. Jung, Eunok Im

**Affiliations:** 1 College of Pharmacy, Pusan National University, Busan 46241, Republic of Korea; 2 Department of Biological Sciences, Oakland University, Rochester, MI 48309, USA; 3 Research Institute for Drug Development, Pusan National University, Busan 46241, Republic of Korea

**Keywords:** atractylodin, *Atractylodes lancea*, PPAR alpha, inflammatory bowel disease

## Abstract

Atractylodin is a major compound in the rhizome of *Atractylodes lancea*, an oriental herbal medicine used for the treatment of gastrointestinal diseases, including dyspepsia, nausea, and diarrhea. Recent studies have shown that atractylodin exerts anti-inflammatory effects in various inflammatory diseases. Herein, we investigated the anti-colitis effects of atractylodin and its molecular targets. We determined the non-cytotoxic concentration of atractylodin (50 μM) using a cell proliferation assay in colonic epithelial cells. We found that pretreatment with atractylodin significantly inhibits tumor necrosis factor-α-induced phosphorylation of nuclear factor-κ-light-chain-enhancer of activated B in HCT116 cells. Through docking simulation analysis, luciferase assays, and *in vitro* binding assays, we found that atractylodin has an affinity for peroxisome proliferator-activated receptor alpha (PPARα). Daily administration of atractylodin (40 mg/kg) increased the survival rate of mice in a dextran sodium sulfate-induced colitis mouse model. Thus, atractylodin can be a good strategy for colitis therapy through inducing PPARα-dependent pathways.

## 1. Introduction

Inflammatory bowel disease (IBD) is a chronic inflammatory disorder of the gastrointestinal tract that is categorized into two major conditions: ulcerative colitis and Crohn’s disease [1]. In recent decades, the incidence of IBD has increased worldwide [2]. IBD has a complex etiology involving multiple factors, such as smoking, diet, stress, and psychological elements [3]. Managing IBD is crucial because long-standing IBD can increase the risk of colorectal cancer by 2–3-fold [4].

The rhizome of *Atractylodes lancea* has been widely used to treat various gastrointestinal diseases, including dyspepsia, flatulence, nausea, and diarrhea in traditional oriental medicine [5]. *Atractylodes lancea* has multiple active compounds, including atractylodin, atractylenolides, and β-eudesmol [6]. Among them, we focused on atractylodin, which has been reported to exert anti-inflammatory effects in various inflammatory diseases. Lipopolysaccharide-induced acute lung injury was ameliorated by atractylodin through inhibition of the nucleotide-binding oligomerization domain-like receptor protein 3 inflammasome and Toll-like receptor 4 pathways [7]. Lipopolysaccharide- and D-galactosamine-induced acute liver failure was also attenuated by atractylodin via suppression of inflammation and oxidative stress [8]. Collagen-induced arthritis was ameliorated and dendritic cell maturation was suppressed by atractylodin [9]. In the process of investigating the effects of atractylodin on the gastrointestinal tract, a study demonstrated the ameliorative effects of atractylodin on intestinal dysmotility, constipation, and diarrhea in an experimental rat model [10]. Recently, atractylodin was reported to ameliorate dextran sodium sulfate-induced colitis in mice by regulating gut microbiota dysbiosis and suppressing inflammation via the mitogen-activated protein kinase (MAPK) pathway [11]. Despite the obvious anti-inflammatory effects of atractylodin, few studies have been conducted on its molecular targets.

Peroxisome proliferator-activated receptors (PPARs) are members of the nuclear receptor superfamily and include several members, including PPARα, PPARβ, and PPARγ. During the past decades, the anti-inflammatory effects of PPARα agonists have been widely studied in various organs. In alveolar epithelial cells, the PPARα agonist WY14643 inhibited lipopolysaccharide-induced inflammation [12]. The PPARα/γ dual agonist MHY966 inhibited the expression of inducible nitric oxide synthase and cyclooxygenase-2 in an ultraviolet B-induced mouse skin inflammation model [13]. In addition, the PPARα agonist MHY553 ameliorated aging-related hepatic steatosis through anti-inflammatory effects [14]. Azuma et al. assessed the anti-inflammatory effects of WY14643 in a dextran sodium sulfate (DSS)-induced colitis mouse model [15]. Administration of WY14643 significantly ameliorated colitis symptoms and reduced the expression levels of inflammatory genes, such as interferon-γ, tumor necrosis factor-α, interleukin-1β, and interleukin-6 in colon tissues.

In the present study, we investigated the anti-inflammatory effects of atractylodin in colonic epithelial cells and in a murine colitis model. Furthermore, we focused on PPARα to elucidate the molecular targets of atractylodin using molecular docking simulation and luciferase assays.

## 2. Results

### 2.1. Effects of Atractylodin on the Viability of Colonic Epithelial Cells

**Figure 1:**
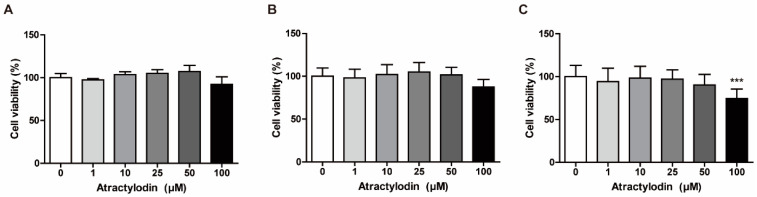
Determination of a non-cytotoxic working concentration of atractylodin in colonic epithelial cells. Cell viability was investigated using an MTT assay. (**A**) NCM460, (**B**) SW480, and (**C**) HCT116 cells were treated with vehicle (0.1% DMSO) or atractylodin at the indicated concentrations for 24 h (n = 6). Viabilities are presented as a percentage of the vehicle-treated group. *** *p* < 0.001 compared to the vehicle-treated group.

Based on the findings of a previous study that investigated the cytotoxic activity of atractylodin in cholangiocarcinoma cells, we investigated the concentration-dependent cytotoxic effects of atractylodin to determine non-cytotoxic concentrations for further experiments [16]. We performed an MTT assay using colonic epithelial cells, including NCM460, SW480, and HCT116 cells. We treated the cells with atractylodin at various concentrations for 24 h to assess cell viability. As depicted in Figure 1, there was a slight decrease in the viability of NCM460 and SW480 cells, but a significant decrease in the viability of HCT116 cells at a concentration of 100 μM. Therefore, in subsequent experiments, we used 50 μM atractylodin, which was the maximal non-cytotoxic concentration in the MTT assay.

### 2.2. Anti-inflammatory Effects of Atractylodin in HCT116 Cells

**Figure 2:**
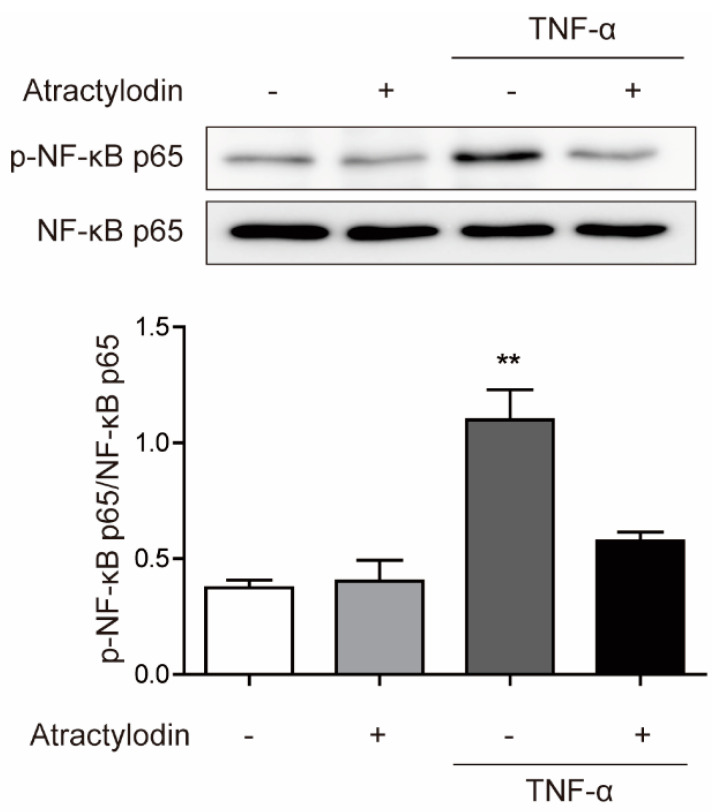
Inhibitory effects of atractylodin against TNF-α-induced NF-κB phosphorylation. HCT116 cells were pretreated with atractylodin (50 μM) for 1 h followed by TNF-α (5 ng/mL) treatment for 15 min. Protein expression were analyzed by immunoblotting analysis. ** *p* < 0.01 compared to the untreated control.

To investigate the anti-inflammatory effects of atractylodin in colonic epithelial cells in vitro, we used HCT116 cells and tumor necrosis factor (TNF)-α. We pretreated atractylodin at a concentration of 50 μM for 1 h and stimulated the cells with TNF-α at a concentration of 5 ng/mL for 15 min. Next, we investigated the phosphorylation of nuclear factor-κ-light-chain-enhancer of activated B (NF-κB) p65, a well-known marker of inflammation [17]. As shown in Figure 2, TNF-α-induced phosphorylation of NF-κB p65 was significantly inhibited by atractylodin pretreatment. These results suggested that atractylodin has anti-inflammatory effects in colonic epithelial cells.

### 2.3. Molecular Docking Studies for Atractylodin and PPARs

As previous studies on the anti-inflammatory effects of atractylodin have seldom identified molecular targets, we focused on PPARs to identify the molecular targets of atractylodin. Molecular docking simulations were employed to determine the binding profile and affinity of atractylodin to ligand-binding domains (LBDs) of PPARα and PPARγ at the molecular level. To validate the docking simulation process, two known PPAR agonists, WY14643 and rosiglitazone, were re-docked onto LBDs of human PPARα (PDB code: 4BCR) and human PPAR-γ (PDB code: 2PRG). Compared to the published co-crystal structures, re-docking of WY14643 and rosiglitazone to hPPARα and hPPARγ LBD, respectively, resulted in the reproduction of binding poses close to the co-crystal structures. Among the nine docking modes ranked by docking scores, the lowest energy scores (−6.3 and −6.3 kcal/mol) were selected as the optimal docking modes of atractylodin to PPARα and PPARγ, respectively.

Unlike rosiglitazone binding to hPPARγ LBD, two molecules of WY14643 bound to the hPPARα LBD in the co-crystal structure. One WY14643 molecule bound to the standard PPAR-α agonist binding site, while an additional molecule bound to a secondary site located between H2′ and H3. Similar to many other PPAR agonists, the aromatic ring of the first WY14643 molecule was located in the hydrophobic region, including residues Cys276, Thr279, Ile317, and Met330, and the carboxyl group formed hydrogen bonds with Ser280 (H3), Tyr314 (H5), His440 (H11), and Tyr464 (H12) of the PPAR-α LBD [18]. The fused heterocyclic rings of the second WY14643 formed a nonpolar interaction with Ile241, Leu247(H2′), Val255, Ile263, Arg271 (H3), Ile272 (H3), His274 (H3), and Cys275 (H3), and the nitrogen and carboxylate groups formed a salt-bridge network with Glu251 (H2′) and Lys266 (ω-loop) and His274, respectively [18]. The pyridine ring of rosiglitazone, located in the hydrophobic pocket of the PPARγ LBD, and the thiazolidinedione group revealed hydrogen bonding interactions with Arg288, Gln286, Ser289 (H3), His323 (H5), His449 (H10), and Tyr473 (H12) [19]. These conserved hydrogen bonding interactions contribute to the stabilization of the AF2-helix (H12) in the active conformation and thus promote the binding of co-activator proteins [18,19,20].

**Figure 3:**
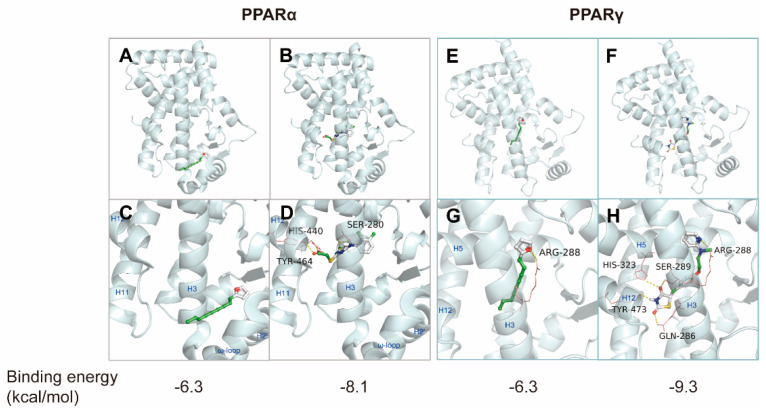
Molecular docking models of atractylodin and PPARα or PPARγ. (**A**,**B**) An overview of the optimal docking modes of atractylodin and WY14643 bound to the ligand binding domain (LBD) of PPARα, respectively. (**C**) Zoomed in view of the docking mode of atractylodin (−6.3 kcal/mol) occupying the ω-loop region of PPARα LBD. (**D**) Zoomed in view of the docking mode of WY14643 (−8.1 kcal/mol) with hydrogen bonds (yellow dotted line) formed with Ser280, His440, and Tyr464 of the PPARα LBD. (**E**,**F**) An overview of the optimal docking modes of atractylodin and rosiglita-zone to the LBD of PPARγ, respectively. (**G**) Zoomed in view of the docking mode of atractylodin (−6.3 kcal/mol) with a hydrogen bond (yellow dotted line) formed with Arg288 of the PPARγ LBD. (**H**) Zoomed in view of the docking mode of rosiglitazone (−9.3 kcal/mol) with hydrogen bonds (yellow dotted line) formed with Ser289, Gln286, His323, Tyr473, and Arg288 of the PPARγ LBD.

The optimal docking mode of atractylodin indicated a lack of hydrogen bonding interactions with key amino acid residues in the PPARα LBD, whereas WY14643 showed a hydrogen bonding pattern corresponding to the co-crystal structure [18] (Figure 3). However, atractylodin occupied the ω-loop region of the PPARα LBD, known as the second binding site, which is enclosed by the amino acid residues Ile241, Leu247, Ala250, Glu251, Leu254, Val255, Leu258, Ile263, Lys266, Arg271, His274, Ala333, and Val332 on helices H3, H2′, and ω-loop (Figure 3). It may be assumed that this binding of atractylodin indirectly stabilizes AF-2 for transactivation. It has been reported that the unique binding mode of WY14643 to the second binding site could induce PPARα activation by non-H12-dependent mechanisms to modulate the stabilization of AF-2, similar to that of the PPARγ partial agonist [18]. 

Regarding atractylodin binding to the PPARγ LBD, the best docking mode showed that the oxygen of the furan ring formed a hydrogen bond with Arg288 on H3, whereas rosiglitazone formed hydrogen bonds with Ser289, Gln286, His323, and Tyr473 in addition to Arg288 (Figure 3). It may be proposed that atractylodin stabilizes H12 in a manner distinct from that of full agonists, such as rosiglitazone. Previous studies have speculated that full agonists may trigger transactivation by directly stabilizing the AF-2 coactivator binding site, whereas partial agonists only stabilize regions away from H12, such as H3 and β-sheets of the PPARγ LBD [21].

Based on these results, atractylodin was suggested as a mild PPARα/γ dual agonist. Atractylodin may be defined as a partial agonist of PPARα/γ, as suggested by the characteristic docking it poses and mild transcriptional activity compared to the full agonists WY14643 and rosiglitazone.

### 2.4. PPARα Agonism of Atractylodin

**Figure 4:**
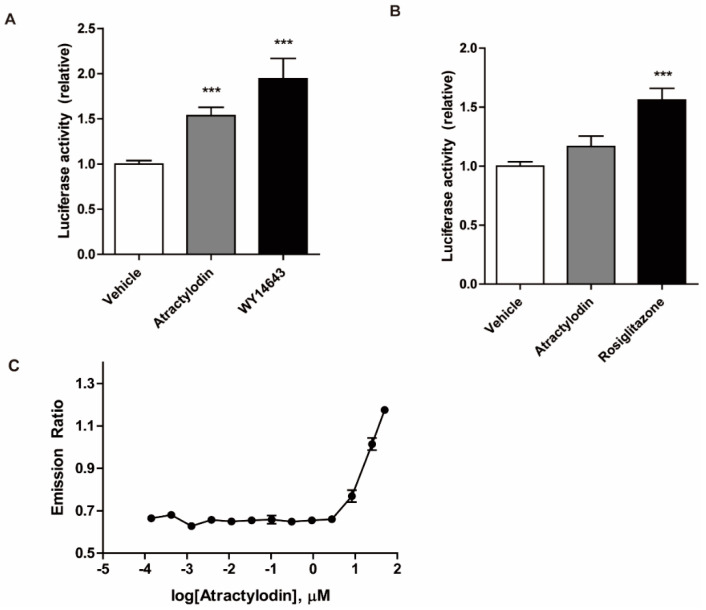
Transcriptional activity of PPARα and PPARγ, and the in vitro ligand-binding activity of PPARα. (**A**,**B**) HCT116 cells were transfected with PPRE and (**A**) PPARα or (**B**) PPARγ. Transfected cells were treated with vehicle (0.1% DMSO), atractylodin (50 μM), WY14643 (10 μM), or rosiglitazone (10 μM) for 5 h. The luciferase activity was detected using a luminometer. Data are presented as a percentage of the vehicle-treated group. *** *p* < 0.001 compared to the vehicle-treated group. (**C**) The binding of atractylodin and PPARα was tested by the time-resolved FRET coactivator assay. Serial dilution of atractylodin (between 0.1 nM and 50 μM), 5 nM PPARα-ligand binding domain, 250 nM fluorescein PGC1a, and 5 nM Tb anti-glutathione-S-transferase antibody were included in the assay composition and incubated for 1 h. The emission ratio (520:495) was calculated and the curves were generated using a sigmoidal dose response equation.

We further investigated the PPAR agonistic effects of atractylodin using a luciferase assay in HCT116 cells. HCT116 cells were transfected with PPRE, PPARα, or PPARγ constructs. The cells were then treated with atractylodin, WY14643, or rosiglitazone. As depicted in Figure 4A,B, atractylodin significantly increased PPARα transcriptional activity. However, despite its binding affinity to PPARγ in the docking simulation, atractylodin did not affect PPARγ transcriptional activity. A potential agonistic effect of PPARα was further investigated by using an in vitro binding assay. As shown in Figure 4C, fluorescence resonance energy transfer (FRET) signal increased by atractylodin in a concentration-dependent manner. These results suggest that atractylodin can be a potential PPARα agonist.

### 2.5. Effects of Atractylodin in a Colitis Mouse Model

**Figure 5:**
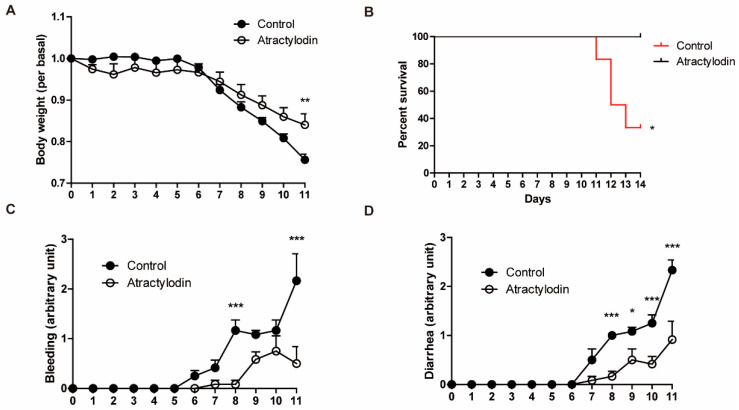
Anti-inflammatory effects of atractylodin in a DSS-induced colitis mouse model. Mice received 2.5% DSS in drinking water for 14 d. Vehicle or atractylodin was administered orally once a day during the DSS administration pe-riod (n = 6). (**A**) Body weight was measured daily and is presented as a relative value of day 0. (**B**) Survival rate was ana-lyzed using the log-rank test (*p* = 0.0209). (**C**,**D**) Clinical symptoms were scored daily on a scale of 0–4. Body weight, bleeding, and diarrhea data were recorded until the first mouse was sacrificed (day 11). * *p* < 0.05, ** *p* < 0.01, and *** *p* < 0.001 compared to the control group.

To investigate the effects of atractylodin in vivo, we utilized a DSS-induced colitis mouse model. DSS was supplied ad libitum with drinking water at a concentration of 2.5% (*w*/*v*). Atractylodin (40 mg/kg) was administered intraperitoneally, concomitant with DSS administration, once daily until the end of the experiment. As shown in Figure 5, colitis symptoms were reduced in atractylodin-treated mice compared to those in vehicle-treated mice. Further, atractylodin-treated mice showed an increased survival rate compared to vehicle-treated mice. These results demonstrated that atractylodin exerts anti-inflammatory effects in vivo. 

## 3. Discussion

In this study, we investigated the anti-inflammatory effects of atractylodin in colitis and the underlying mechanisms, both in vivo and in vitro. Consistent with previous reports demonstrating the anti-inflammatory effects of atractylodin in numerous inflammatory models, we observed clear anti-inflammatory effects of atractylodin against TNF-α-induced cellular inflammation and DSS-induced colitis in mice. Phosphorylation of NF-κB p65, a well-known inflammatory marker, was reduced by atractylodin pretreatment in TNF-α-treated HCT116 cells (Figure 2). This was further investigated using an animal model. In the DSS-induced colitis mouse model, disease symptoms, including body weight loss, rectal bleeding, and diarrhea, were ameliorated by the daily oral administration of atractylodin (Figure 5). These results provided scientific evidence for the classical use of *Atractylodes lancea* in treating gastrointestinal disorders [5]. In addition, these results highlighted the therapeutic advantage of atractylodin, as a single compound, rather than a crude extract, which may have various unidentified constituents.

Furthermore, although several studies have reported the anti-inflammatory effects of atractylodin, few studies have suggested the molecular targets of atractylodin. Recently, it was suggested that the anti-inflammatory effects of atractylodin are mediated by the MAPK and nucleophosmin-anaplastic lymphoma kinase (NPM-ALK) signaling pathways [11,22]. Moreover, phosphorylation of MAPKs (extracellular signal-regulated kinase, p38) can modulate PPARa and PPARg [23]. In IBD, PPARα activation represses NF-κB signaling, which decreases the inflammatory cytokine production by different cell types, whereas PPARγ ligands can inhibit activation of macrophages and the production of inflammatory cytokines, such as TNF-α, interleukin (IL)-6, and Il-1β [24]. However, these studies did not identify an upstream molecule that interacted with atractylodin. Therefore, it is feasible approach that anti-inflammatory effect of atractylodin is related to PPAR modulation. In our study, we identified the molecular targets of atractylodin. We found the agonistic effects of atractylodin on PPARα in two different ways: molecular docking simulation in silico and luciferase assays in vitro. As there have been many studies on the anti-inflammatory effects of PPARα agonists, and as the PPARα pathway also includes the MAPK pathway, it is highly probable that atractylodin attenuates inflammation via PPARα agonistic effects.

Our findings not only suggested the anti-inflammatory effects and the target of atractylodin itself but also the possibility of identifying novel drug candidates. Although the result of an in silico docking experiment suggests a possible interaction between atractylodin and the LBD of PPARα (Figure 3), this does not sufficiently infer a direct interaction. In addition, we compared the luciferase activity of atractylodin with those of the well-known PPARα agonist WY14643 (Figure 4). Increased luciferase activity by atractlyodin suggests that atractylodin regulates PPARα either directly or indirectly. It is possible that atractylodin may stimulate other anti-inflammatory molecules that can regulate PPARα activity. Further studies are warranted to analyze an interaction between PPAR and atractylodin. Using the TR-FRET PPARα coactivator assay, the binding of atactylodin and PPARα was observed suggesting that atractylodin as a potential PPARα agonist. Moreover, there are several studies elaborating in vitro direct ligand-protein binding experiments. A homogeneous fluorescence polarization ligand binding assay can identify ligands that bind to both PPARα and PPARγ using a fluorescein-labeled analog of a potent dual PPARα/γ activator and purified PPARα or PPARγ ligand binding domains [25]. Another study utilized combinational approaches to elucidate the interaction between the selective PPARα modulator pemafibrate and PPARα [26]. Those structural and functional analysis approaches including X-ray crystallography, isothermal titration calorimetry, and fragment molecular orbital analysis precisely elucidated the molecular mechanism behind the binding of pemafibrate and PPARα. Therefore, we could utilize these combinational approaches to further investigate the interaction between atractylodin and PPARα.

In conclusion, we confirmed both the anti-inflammatory effects and the molecular targets of atractylodin. Atractylodin shows anti-inflammatory effects in colitis by activating PPARα, which was supported by in silico, in vitro, and in vivo data. Furthermore, the chemical structure of the molecule can be optimized for improved therapeutics by derivatization in future studies.

## 4. Materials and Methods

### 4.1. Cell Culture

NCM460, a normal human colonic epithelial cell line, was obtained from the INCELL Corporation (San Antonio, TX, USA). The human colorectal cancer cell lines SW480 and HCT116 were purchased from the Korean Cell Line Bank (Seoul, Republic of Korea). NCM460 cells were cultured in DMEM/high-glucose medium (HyClone, Logan, UT, USA). SW480 and HCT116 cells were cultured in RPMI (HyClone). All culture media were supplemented with 10% heat-inactivated fetal bovine serum, 100 units/mL penicillin, and 100 μg/mL streptomycin (HyClone). All the cells were incubated at 37 °C in a 5% CO_2_ atmosphere.

### 4.2. Cell Viability Assay

The MTT assay was performed as previously described [27]. MTT solution was prepared by dissolving thiazolyl blue tetrazolium bromide (Sigma-Aldrich, St. Louis, MO, USA) in phosphate-buffered saline (HyClone) to obtain a stock solution at a concentration of 5 mg/mL and further diluted with culture medium to a working concentration of 0.5 mg/mL. The cells were treated with vehicle or atractylodin at various concentrations for 24 h. The cells were then incubated in MTT solution for 2 h in the dark. After the removal of the MTT solution, DMSO was added to dissolve the formazan crystals. Absorbance was measured at 540 nm using a microplate spectrophotometer (Thermo Fisher Scientific, Waltham, MA, USA).

### 4.3. Immunoblotting Analysis

Total protein was extracted from cells using a protein extraction solution (ELPIS Biotech, Daejeon, Republic of Korea). The protein concentration of the lysates was determined using the Pierce BCA Protein Assay Kit (Thermo Fisher Scientific) according to the manufacturer’s instructions. Equal amounts of proteins from each group were separated by SDS-PAGE and then transferred onto PVDF membranes (Merck KGaA, Darmstadt, Germany). Membranes were incubated with blocking solutions for 1 h and subsequently incubated with primary antibodies overnight at 4 °C. They were then incubated with secondary antibodies for 1 h at room temperature. The bands were visualized using an enhanced chemiluminescence solution (Advansta, San Jose, CA, USA) and ChemiDoc^TM^ Touch Gel Imaging System (Bio-Rad Laboratories, Hercules, CA, USA).

### 4.4. Molecular Modeling

Docking calculations were performed using Auto Dock Vina 1.1.2 software with default settings, and the scoring function of Vina was applied. For ligand preparation, Chem3D Ultra 8.0 software was used to convert the 2D structures of the candidates into 3D structural data. Protein coordinates were downloaded from the Protein Data Bank (accession codes PPARα:4BCR, PPARγ:2 PRG). Chain A was prepared for docking within the molecular modeling software package Chimera 1.5.3 by removing other chains, ligands, and water molecules and by calculating the protonation state of the protein. The addition of polar hydrogen and setting of the grid box parameters were performed using MGLTools 1.5.6. PyMol v1.5 was used to analyze and visually investigate the ligand-protein interactions of the docking.

### 4.5. Luciferase Assay

The luciferase assay was performed as previously described [28]. HCT116 cells were transfected with the PPRE plasmid plus PPARα or PPARγ expression vector using Lipofectamine 3000 reagent (Invitrogen, Waltham, MA, USA) according to the manufacturer’s instructions. All constructs were obtained from Dr. Hae Young Chung (Pusan National University, Busan, Republic of Korea). After transfection, cells were treated with vehicle, atractylodin, WY14643, or rosiglitazone for 5 h. Luciferase activity was measured using the One-Glo Luciferase Assay System (Promega, Madison, MI, USA) and a LuBi microplate luminometer (Seoulin Bioscience, Seongnam, Republic of Korea).

### 4.6. Animal Model

Male C57BL/6 mice (eight weeks old) were obtained from Samtako Bio Korea (Osan, Republic of Korea). The animals were housed under a 12 h light/dark cycle and fed standard rodent chow (Samtako Bio Korea) *ad libitum*. Prior to the experiment, the mice were acclimated for a week. Dextran sodium sulfate (MP Biomedicals, Irvine, CA, USA) was dissolved in tap water at a concentration of 2.5% (*w/v*) and supplied to the mice *ad libitum* for 14 d. Concomitant with DSS administration, vehicle or atractylodin (40 mg/kg) was administered orally once a day. Body weights and clinical symptoms were monitored daily. The degree of bleeding and diarrhea was scored on a scale of 0–4. The study protocols used in our study were reviewed and approved by the Institutional Animal Care and Use Committee of Pusan National University.

### 4.7. TR-FRET-Based PPARα Binding Assay

The interaction of atractylodin and PPARα was investigated using LanthaScreen time-resolved (TR-FRET) PPARα Coactivator assays kits (Invitrogen, Carlsbad, CA, USA), following the manufacturer’s instructions. Briefly, PPARα ligand binding domain was added to serial dilution of atractylodin (between 0.1 nM and 50 μM), a terbium-labeled anti-glutathione-S-transferase antibody, and a fluorescence-labeled PGC1a coactivator peptide. After 1 h incubation at room temperature, the FRET signal was measured at wavelengths of 520 nm and 495 nm by a fluorescence multi-well plate reader (TriStar LB 941; Berthold Technologies, Bad Wildbad, Germany. The TR-FRET ratio (520:495) was calculated and a binding curve was generated by plotting the emission ratio vs. the log [atractylodin].

### 4.8. Statistical Analysis

Statistical analyses were conducted using GraphPad Prism 5 (GraphPad Software, San Diego, CA, USA). Results are presented as mean ± standard error. Data were analyzed using a t-test one-way analysis of variance followed by Tukey’s test.

## Data Availability

Not applicable.
